# Fast- or Slow-inactivated State Preference of Na^+^ Channel Inhibitors: A Simulation and Experimental Study

**DOI:** 10.1371/journal.pcbi.1000818

**Published:** 2010-06-17

**Authors:** Robert Karoly, Nora Lenkey, Andras O. Juhasz, E. Sylvester Vizi, Arpad Mike

**Affiliations:** Department of Pharmacology, Institute of Experimental Medicine, Hungarian Academy of Sciences, Budapest, Hungary; University of Toronto, Canada

## Abstract

Sodium channels are one of the most intensively studied drug targets. Sodium channel inhibitors (e.g., local anesthetics, anticonvulsants, antiarrhythmics and analgesics) exert their effect by stabilizing an inactivated conformation of the channels. Besides the fast-inactivated conformation, sodium channels have several distinct slow-inactivated conformational states. Stabilization of a slow-inactivated state has been proposed to be advantageous for certain therapeutic applications. Special voltage protocols are used to evoke slow inactivation of sodium channels. It is assumed that efficacy of a drug in these protocols indicates slow-inactivated state preference. We tested this assumption in simulations using four prototypical drug inhibitory mechanisms (fast or slow-inactivated state preference, with either fast or slow binding kinetics) and a kinetic model for sodium channels. Unexpectedly, we found that efficacy in these protocols (e.g., a shift of the “steady-state slow inactivation curve”), was not a reliable indicator of slow-inactivated state preference. Slowly associating fast-inactivated state-preferring drugs were indistinguishable from slow-inactivated state-preferring drugs. On the other hand, fast- and slow-inactivated state-preferring drugs tended to preferentially affect onset and recovery, respectively. The robustness of these observations was verified: i) by performing a Monte Carlo study on the effects of randomly modifying model parameters, ii) by testing the same drugs in a fundamentally different model and iii) by an analysis of the effect of systematically changing drug-specific parameters. In patch clamp electrophysiology experiments we tested five sodium channel inhibitor drugs on native sodium channels of cultured hippocampal neurons. For lidocaine, phenytoin and carbamazepine our data indicate a preference for the fast-inactivated state, while the results for fluoxetine and desipramine are inconclusive. We suggest that conclusions based on voltage protocols that are used to detect slow-inactivated state preference are unreliable and should be re-evaluated.

## Author Summary

Sodium channels are the key proteins for action potential firing in most excitable cells. Inhibitor drugs prevent excitation (local anesthetics), regulate excitability (antiarrhythmics), or prevent overexcitation (antiepileptic, antispastic and neuroprotective drugs) by binding to the channel and keeping it in one of the inactivated channel conformations. Sodium channels have one fast- and several slow-inactivated conformations (states). The specific stabilization of slow-inactivated states have been proposed to be advantageous in certain therapeutic applications. The question of whether individual drugs stabilize the fast or the slow-inactivated state is studied using specific voltage protocols. We tested the reliability of conclusions based on these protocols in simulation experiments using a model of sodium channels, and we found that fast- and slow-inactivated state-stabilizing drugs could not be differentiated. We suggested a method by which the state preference of at least a subset of individual drugs could be determined and tried the method in electrophysiology experiments with five individual drugs. Three of the drugs (lidocaine, phenytoin and carbamazepine) were classified as fast-inactivated state-stabilizers, while the state preference of fluoxetine and desipramine was found to be undeterminable by this method.

## Introduction

Sodium channels are the key proteins in action potential firing for most excitable cells. They exhibit a complex, membrane potential-dependent gating behavior [1]. Even minor disturbances in the gating behavior can lead to hyperexcitability, which can be one of the causes of various disorders such as epilepsy, migraine, neuropathic and inflammatory pain, muscle spasms, and chronic neurodegenerative diseases. For several decades, sodium channel inhibitors (SCIs) have been successfully used to lower excitability as, for example, local anesthetics, anticonvulsants, antiarrhythmics, analgesics, antispastics and neuroprotective agents. Interestingly, the majority of antidepressants were also found to be potent SCIs. In a recent study [2] the highest incidence of SCI activity was found amongst this therapeutic class. We intend to test if the mechanism of action on sodium channels is similar to that of classic SCIs.

Thus far only a single drug binding site is established unequivocally on sodium channels, the “local anesthetic receptor”, located within the inner vestibule, its key residue being the phenylalanine located right below the selectivity filter, on domain 4 segment 6 [3]. However, the contribution of individual residues within the inner vestibule changes from drug to drug [4]–[6]. For certain drugs an alternative binding site have been proposed, which is supposed to be located within the outer pore [7], [8], but the exact position of the binding site(s) for specific SCIs (other than local anesthetics) is currently unsettled. For our case the exact location of the binding site is not relevant, we only need to suppose that the major mechanism of inhibition is preferential affinity to-, and stabilization of a specific inactivated state.

The major mechanism of SCIs is stabilization of an inactivated channel conformational state as a result of a preferential affinity for that state. The question of which inactivated state is preferred is under debate for many SCI drugs (e.g. [9]–[12], or [13]–[17]). Sodium channels are capable of fast inactivation (complete within a few milliseconds), and different forms of slow inactivation (time constants ranging from ∼100 ms to several minutes) [18]. Slow-inactivated state preference has been proposed as a therapeutic advantage [19]–[21]. Mutations of sodium channel genes which affect slow inactivation are associated with several diseases [22]. Slow inactivation determines sodium channel availability, and thereby contributes to overall membrane excitability, determining the propensity to generate repetitive firing, and the extent of action potential backpropagation. Slow inactivated state preference has been proposed as a potential therapeutic advantage in specific types of epilepsy, neuropathic pain and certain arrhythmias [19]–[22]. Furthermore, this mechanism of sodium channel inhibition has been proposed to modulate neuronal plasticity [22]. In recent years a number of novel slow-inactivated state-preferring drug candidates have been described, including the recently approved antiepileptic drug lacosamide (Vimpat) [19], [23]. This drug has been found to be effective in a model of treatment-resistant seizures, and of diabetic neuropathic pain, in which tests conventional anticonvulsants were found ineffective [19].

Special voltage protocols are used to evoke and study the slow-inactivated state. Availability of channels is studied after a prolonged depolarization (to induce slow inactivation), followed by a hyperpolarizing gap (to allow recovery from fast, but not slow inactivation). Because availability in such protocols is solely determined by the extent of slow inactivation, a drug that decreases availability is considered to be slow-inactivated state-preferring. However, gating rates (the rate of inactivation and rate of recovery from inactivation) are altered by drug binding. A fast-inactivated state-preferring drug stabilizes this state by delaying recovery. A delayed recovery does not necessarily indicate actual modification of the gating rate. For example if the bound drug prevents recovery from inactivation, then recovery will appear to be slowed because the drug needs first to dissociate [24], [25]. In our current study, however, we chose to use a model according to the modulated receptor hypothesis [26], [27], i.e., the change in affinity equals the actual modification of the gating rates. For this reason in our model increased affinity is synonymous with state stabilization. Altered gating rates have been experimentally demonstrated using gating charge measurements [28], [29]. Because of the altered gating, the rate of recovery from fast inactivation in the presence of the drug can easily overlap with the rate of recovery from slow-inactivated state. The rate of state-dependent association and dissociation of the drug should also be taken into account. As a result, interpretation of data obtained with these protocols is not straightforward (e.g. [9], [30]).

With the help of simulations, we intended to understand the interactions between binding and gating rates and wanted to test the major prototypical inhibitor mechanisms in commonly used protocols. We wanted to explore what could be deduced from these data, and wanted to find the right protocols that could help to determine the inhibition mechanisms.

**Figure 1:**
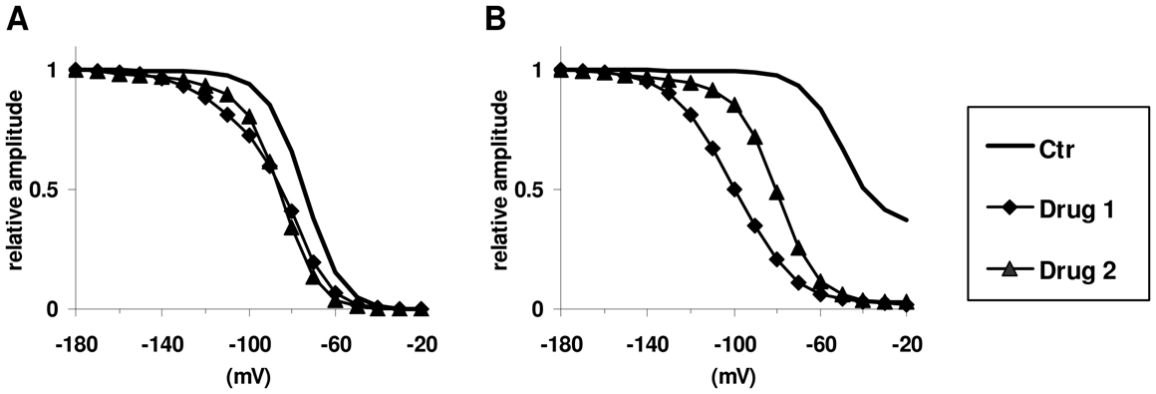
Effectiveness of two simulated drugs in “steady-state fast inactivation” and “steady-state slow inactivation” protocols. A typical example showing that state preference of drugs cannot reliably be deduced from these protocols (see Figure 2). Drug 1 has preferential affinity to fast inactivated state, while Drug 2 prefers slow inactivated state. **A**, “steady-state fast inactivation” protocol. **B**, “steady-state slow inactivation” protocol.

**Figure 2:**
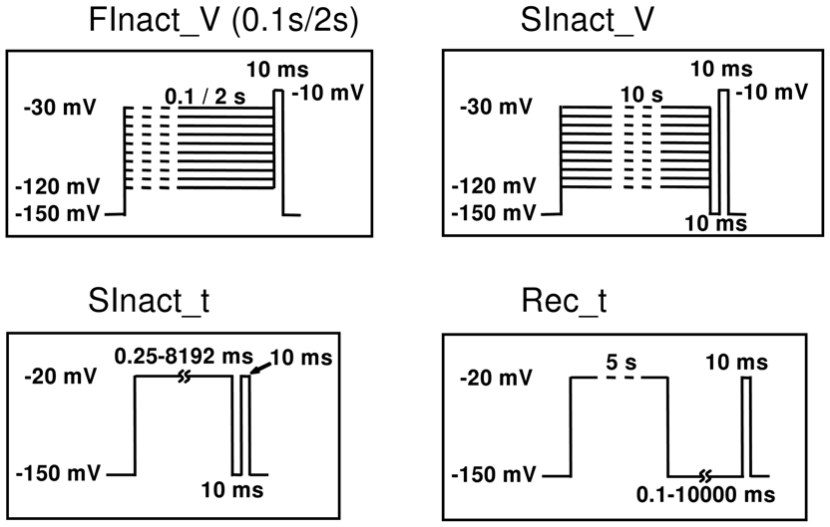
Protocols used in experiments and simulations. For explanation see text.

Our data suggest that conclusions based on conventional protocols are not reliable. For example, the fact that one drug preferentially shifts the “steady-state slow inactivation curve” as compared to another drug does not necessarily mean that the drug prefers the slow-inactivated state. Figure 1 illustrates two (simulated) drugs investigated in “steady-state inactivation” protocols (protocols are discussed below). Both drugs shifted the “fast inactivation curve” (Figure 2, “FInact_V”) to the same degree, but Drug 1 caused a larger shift in the “slow inactivation curve” (Figure 2, “SInact_V”). In this special case, however, Drug 1 was defined to have a higher affinity to fast inactivated state, while Drug 2 had a higher affinity to slow inactivated state. We observed, on the other hand, that fast- and slow-inactivated state-preferring drugs tended to preferentially affect the onset of inactivation and recovery, respectively. Therefore we combined the information from these two protocols by plotting effectiveness in one protocol as a function of effectiveness in the other. We observed that data points for fast- and slow-inactivated state-preferring drugs were confined to definite areas of the effectiveness (inactivation) – effectiveness (recovery) plane. The two areas were found to overlap; therefore, explicit determination of the mechanism was not possible in all cases.

Using patch-clamp experiments, we tested three classic SCIs (lidocaine, phenytoin and carbamazepine) and two antidepressants (fluoxetine and desipramine). Properties of inhibition by classic SCIs were consistent with fast-inactivated state preference with fast binding kinetics. Inhibition by antidepressants was distinctly different. Whether the difference was caused by slow binding kinetics or slow-inactivated state preference could not be determined.

## Results

### Models

For simulations two different kinds of models were used: a phenomenological Hodgkin-Huxley type model and a state model similar to the one published by Kuo and Bean [31]. In both models, however, we introduced slow-inactivated states and drug-bound states with altered gating transition rates. For a detailed description of the models see Methods and Text S1. The Hodgkin-Huxley type model, which will be referred to as the “tetracube” model because of its topology (see Methods), was used for most simulations. The Kuo-Bean type model, referred to as the “multi-step-activation” (MSA) model, was only used for testing the robustness of our observations. In the models, both the degree of alteration of the transition rates and the state preference (the difference between affinities for different states) were given by a single factor *CF* (for fast-inactivated state-preferring drugs) or *CS* (for slow-inactivated state-preferring drugs). The kinetics of association and dissociation to the resting state are defined by the rate constants *k_a_* and *k_d_*, respectively. Association and dissociation rate constants to other states were calculated as described in Methods.

### Test protocols

To compare simulated data with experimental results, we used similar voltage protocols in both the simulations and experiments (Figure 2). Throughout this study we used four protocols:

“**FInact_V**” is a standard “steady-state fast inactivation” protocol in which availability is assessed as a function of pre-pulse membrane potential. The pre-pulse duration was 0.1 s when we compared the effects of “FInact_V” and “SInact_V”. In electrophysiology experiments, because drugs with differing mechanisms of action and association kinetics had to be compared, a 2 s pre-pulse duration was used. Note, that although we use the widespread term “steady-state fast inactivation” protocol, the term is incorrect for two reasons. First, it is not necessarily “steady-state” in the sense that the pre-pulse duration may not be long enough for reaching equilibrium of either drug binding or channel gating (2 s is enough for the development of some degree of slow inactivation). Second, “availability” would be a better term than “inactivation”, because the protocol does not necessarily reflect only inactivation in the presence of a drug, since we cannot separate blocked open and inactivated channels; however, “fast availability” and “slow availability” protocols are improper terms.

“**SInact_V**” is a “steady-state slow inactivation” protocol in which occupancy of the slow-inactivated state is intended to be measured as a function of the membrane potential. It differs from the previous protocol in two respects: pre-pulse duration is longer (10 s), allowing more complete development of slow inactivation; and this protocol contains a 10 ms hyperpolarizing (−150 mV) gap between the pre-pulse and the test pulse. The hyperpolarizing gap serves to separate occupancy of the slow-inactivated state from that of fast-inactivated states: >95% of channels recover from the fast-inactivated state within this period. Despite the name, however, neither “FInact_V” nor “SInact_V” is able to measure drug effects on a pure population of fast or slow-inactivated channels. Fast inactivation practically reaches equilibrium at most membrane potentials within ∼10 ms. With longer durations of pre-pulses in the “FInact_V” protocols the ratio of slow-inactivated channels increases from ∼5% (0.1 s pre-pulse) to ∼40% (2 s pre-pulse). This is accompanied by a minor shift of the curve (ΔV_1/2_<4 mV). Drug effects can further change this distribution depending on binding kinetics and state preference. In “SInact_V” protocols most unavailable channels are in a slow-inactivated state in the absence of drugs. However, the presence of a drug may alter the distribution of channel states. The unavailable fraction does not consist of slow-inactivated channels only but also is “contaminated” with drug-bound fast-inactivated channels. The conventional name “steady-state” therefore is absolutely untrue for this protocol, as the extent and V_1/2_ of slow inactivation is strongly dependent on pre-pulse duration. We nevertheless need to use this terminology as we have discussed above.

“**SInact_t**” (“Slow inactivation onset as a function of time”) monitors the effect of prolonged depolarizations on sodium channel availability. In the absence of drugs, the onset of slow inactivation is monitored as a function of time (duration of depolarizing pulses). In the presence of a drug, it is not clear whether it reflects pure slow inactivation or a mixture of fast and slow inactivation (see below for a detailed explanation).

“**Rec_t**” (“Recovery from inactivation as a function of time”) monitors recovery after a 5 s depolarization to −20 mV as a function of hyperpolarizing gap duration (the gap is between the 5 s pre-pulse and the test pulse). In the absence of drugs, a 5 s depolarization causes both fast and slow inactivation (approximately 45–55%, respectively), and the protocol monitors recovery from both states. The time constants for recovery were 2.21 and 58.25 ms [32]. In the presence of drugs, measured recovery reflects the combination of dissociation and recovery from both inactivated states.

Concentration-response curves were simulated using single depolarizations to 0 mV from holding potentials of −150, −90 and −60 mV.

### Simulation experiments: Four prototypical mechanisms

#### Defining the prototypical mechanisms

In order to address the problem of fast- vs. slow-inactivated state preference and the interaction between the dynamics of binding and gating, we simulated four prototypical mechanisms: either the fast- or the slow-inactivated state was preferred by the drug (“FI” and “SI”, respectively), and the drug had either fast or slow binding kinetics (“fb” and “sb”, respectively). Fast-inactivated state preference was introduced by setting *CF* = 10 (with *CS* = 1). Slow-inactivated state-preferring drugs were defined by *CS* = 10 (*CF* = 1). Association and dissociation rate constants for drugs with fast binding kinetics were set to the values: *k_a_* = 0.5 s^−1^µM^−1^ and *k_d_* = 100 s^−1^, while slow binding kinetics were defined as *k_a_* = 0.005 s^−1^µM^−1^ and *k_d_* = 1 s^−1^. The term “fast” is relative, of course. All SCIs associate relatively slowly compared to hydrophilic drugs that bind to a readily accessible extracellular site. We chose these values so that they would be close to the rate constants that had been determined in previous studies for classic sodium channel inhibitors (e.g. [12]). Combinations of these properties give the four prototypical “drugs.” Current hypotheses for the mechanism of antidepressant action are represented by either the drug “FI_sb” (fast-inactivated state is stabilized, with slow binding kinetics) or the “SI_fb” drug (slow-inactivated state is stabilized, and binding kinetics are not rate limiting). A third hypothesis for the mechanism of antidepressants is also evaluated because it is also possible that both slow-inactivated state preference and slow binding kinetics determine the onset rate of inhibition (“SI_sb”). Inhibition by anticonvulsants has a rapid onset, but the identity of the preferred state is debated. Alternative anticonvulsant hypotheses, therefore, are represented by either “SI_fb” or “FI_fb.”

#### Slow association and slow inactivation preferences could not be discriminated by conventional voltage protocols

**Figure 3:**
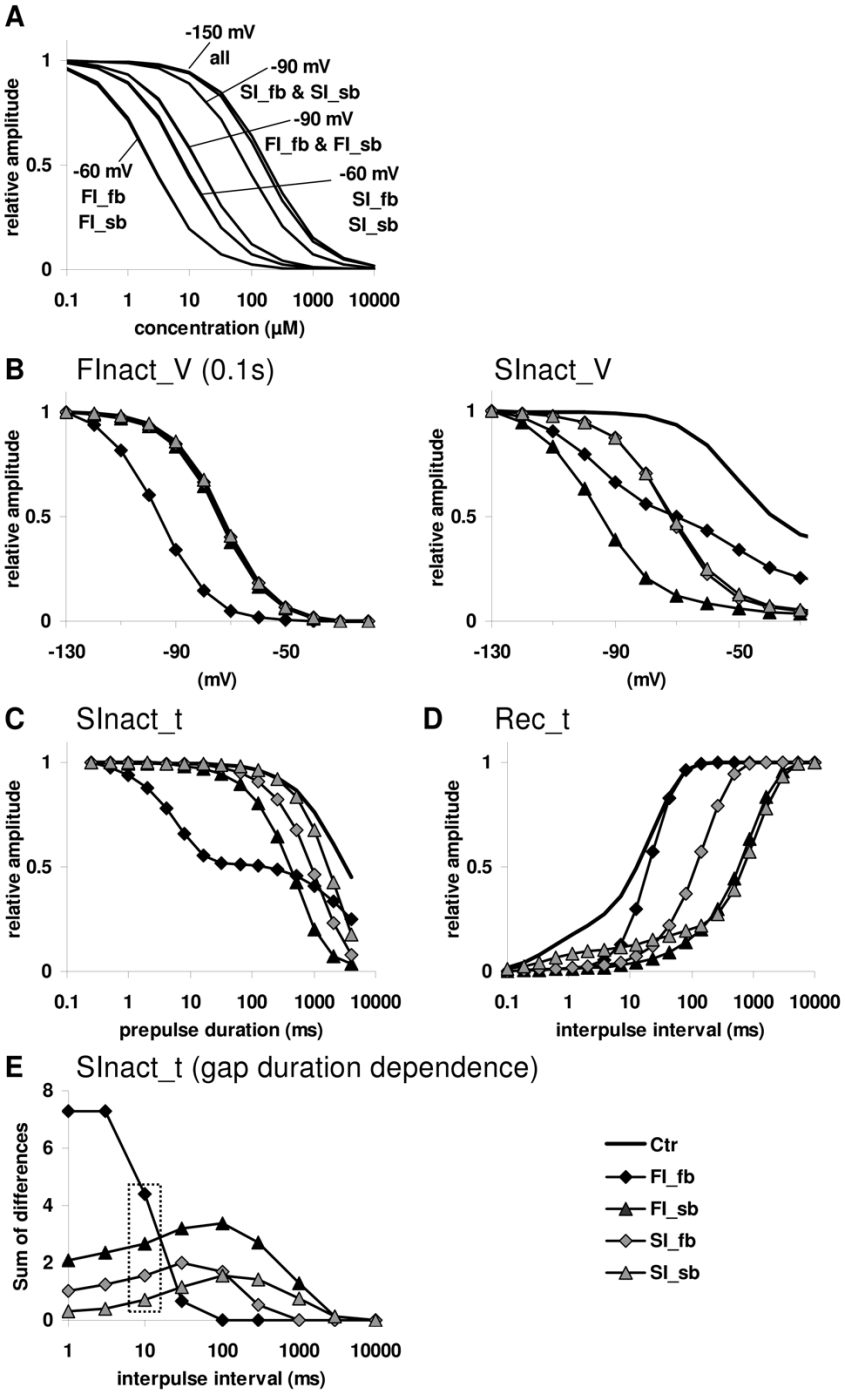
Results of simulations with the tetracube model using the four prototypical mechanisms. The mechanisms were: “FI_fb” (fast-inactivated state is stabilized, with fast binding kinetics), “FI_sb” (fast-inactivated state is stabilized, with slow binding kinetics), “SI_fb” (slow-inactivated state is stabilized, with fast binding kinetics) and “SI_sb” (slow-inactivated state is stabilized, with slow binding kinetics). **A**, Concentration response curves. **B**, Effect of simulated “drugs” on “steady-state fast inactivation” (left panel) and “steady-state slow inactivation” (right panel) protocols. **C**, Effect of simulated “drugs” on the slow inactivation onset curve. **D**, Effect of simulated “drugs” on the recovery curve. **E**, Dependence of the effect of simulated “drugs” on the duration of the hyperpolarizing gap. The effect was quantified by calculating the areas between curves from the semilogarithmic plots. Box indicates data calculated from curves seen in Figure 3C.

Simulations with the tetracube model, obtained under control conditions and in the continuous presence of 30 µM of any of the four prototypical “drugs,” are shown in Figure 3 A–D.

No difference in the concentration-response curves between the drugs with fast and slow association rates was found at very negative holding potentials (−150 mV), at which there is no significant inactivation (Figure 3A). At less hyperpolarized membrane potentials (−90 and −60 mV), the relative potency of drugs was model parameter dependent, determined by the fraction of inactivated channels: When the *V_1/2_* value for slow inactivation was more negative, “SI” drugs were more potent (data not shown). When *V_1/2_* for fast inactivation was more negative (which was the case when we used the particular parameters obtained by optimization – Table S3), “FI” drugs seemed to be more potent (Figure 3A). Binding kinetics had minor influence on concentration response curves.

If a drug has no effect on the “steady-state fast inactivation curve”, but it shifts the “steady-state slow inactivation” curve substantially, one might think that the reason for this must be slow-inactivated state preference. To test this argument, we simulated both “steady-state fast inactivation” and “steady-state slow inactivation” curves. For the “FInact_V” curve, a 100 ms long conditioning pulse (ranging from −120 mV to −30 mV) preceded the test pulse (Figure 3B left panel). For the slow inactivation curve, the duration of the conditioning pulse was extended to 10 s, and a 10 ms hyperpolarization to −150 mV was inserted before the test pulse to allow recovery from the fast-inactivated state (Figure 3B right panel). The only drug that shifted the “steady-state fast inactivation” curve appreciably was “FI_fb.” “FI_sb” failed to affect this curve because its association was not fast enough to act significantly within the time of the pre-pulse (100 ms). The slow inactivation curve was shifted by all four drugs. The protocol, thus, failed to distinguish slow-inactivated state-preferring and slowly associating drugs.

In the “SInact_t” protocol, the four drugs shifted the curve in clearly dissimilar patterns (Figure 3C). Unexpectedly, fast-inactivated state-stabilizing drugs caused a larger shift of the “slow inactivation curve” than slow-inactivated state-preferring drugs. The reason for this counter-intuitive behavior is that, once the recovery from the fast-inactivated state is slowed down, the curve no longer reflects the ratio of slow-inactivated channels, but instead reflects a mixture of slow and fast-inactivated channels. The lack of effect on the process of slow inactivation can be seen in the biphasic pattern of the curve in the presence of drug “FI_fb.” This pattern is remarkably similar to the curve obtained experimentally with 300 µM lidocaine or 300 µM carbamazepine using the protocol with the 5 ms hyperpolarizing gap (see below).

In the “Rec_t” protocol, “FI_fb” behaved as expected, affecting only the part of the curve that is responsible for recovery from the fast-inactivated state. However, drug “FI_sb” was as effective as slow-inactivated state-preferring drugs. In experiments using this protocol, the recovery from inhibition is slowed down for two reasons: i) the slow dissociation rate of the drug; and ii) drug-bound channels display a slowed gating. In the case of “FI_sb,” the former seems to be the rate-limiting step and, if dissociation is slow enough, the curve is shifted, whichever state is preferred (Figure 3D).

Although recovery from fast inactivation is almost complete (95%) within the 10 ms gap, some studies used longer gaps (100 ms to 1 s) in “steady-state slow inactivation” (“SInact_V”) or onset of slow inactivation (“SInact_t”) protocols in order to make sure that even drug-bound fast-inactivated channels had time to recover. However, this time is still insufficient for drugs with slow dissociation kinetics. To test how the relative potency of the four drugs depends on gap duration, we made simulations with different gap durations ranging from 1 ms to 10 s. The curves shown in Figure 3C were generated using different gap durations. The potency of the drugs was quantified by calculating the sum of differences between control and drug curves, and the values were plotted against gap duration in Figure 3E. While the effect of drug “FI_fb” disappeared at gap durations above 100 ms, “FI_sb” remained more potent than “SI” drugs throughout the range of durations.

In summary, conventional voltage protocols failed to reliably distinguish between drugs “FI_sb” and “SI_fb.”In particular, results of the “SInact_t” protocol are not only unreliable, but are clearly misleading as “FI_sb” caused a much larger shift than “SI_fb.”

#### Test of robustness #1: Simulations with the MSA model

We made three major observations in the simulations described above: 1) A shift of the “SInact_V” curve does not necessarily reflect a slow-inactivated state preference; 2) Fast-inactivated state-stabilizing drugs caused a larger shift of the “SInact_t” curve than slow-inactivated state-preferring drugs; and 3) Slow-inactivated state-preferring drugs are not necessarily more effective than fast-inactivated state-preferring ones in delaying recovery in the “Rec_t” protocol.

In order to judge the reliability of these observations, we first repeated the experiments using the MSA model, an allosteric model where inactivation processes draw their voltage-dependencies from the voltage-dependence of the activation process. Results of the simulations are shown in Figure S2. Although simulation results with the MSA model gave somewhat different results (see Text S1), the three major observations were evidently confirmed.

#### Test of robustness #2: Simulations with the tetracube model with random parameters

**Figure 4:**
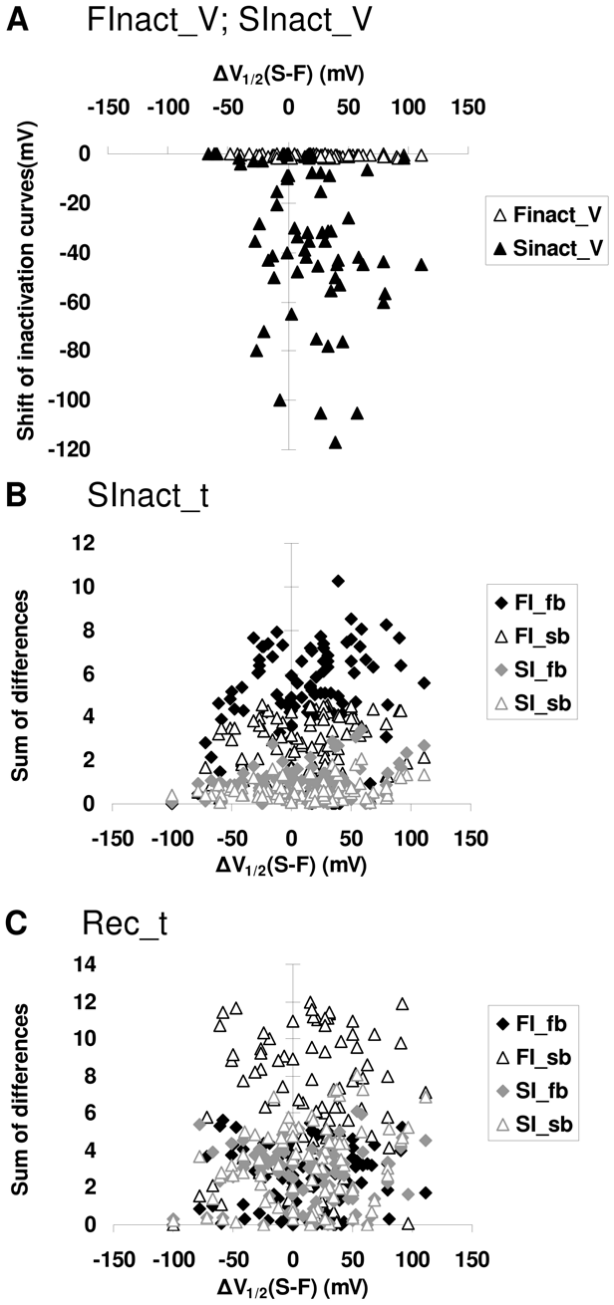
Results of 100 simulations with random parameters of the tetracube model. Measures of the potency of the four prototypical “drugs” are plotted against the difference between *V_1/2_* values for fast and slow inactivation. **A**, Leftward shift of the V_1/2_ of “FInact_V” and “SInact_V” curves, caused by “FI_sb.” **B**, Effect of all four “drugs” on the “SInact_t” curve. The effect was quantified by calculating the sum of differences between control and drug curves (see Figure 3C). **C**, Effect of all four “drugs” on the “Rec_t” curve. Sum of differences between the curves in control conditions, and during drug application. (see Figure 3D).

We also tested the consistency of our observations by varying parameters of the tetracube model. We wanted to test at which part of the parameter space they were true and under what conditions they failed. To this end, we performed Monte Carlo simulations. All 18 parameters were randomized between the constraints shown in Table S3. With each set of randomized parameters the following protocols were simulated: 1) “SInact_V” using drug “FI_sb;” 2) “SInact_t” using all four drugs; and 3) “Rec_t” using all four drugs. One hundred individual sets of random parameters were generated. Most of these produced channels with abnormal voltage-dependences or kinetics of gating. (We only constrained parameters for the rate constants. The kinetics and equilibrium of any of the three gates, however, are determined by six parameters. Even if each of the six parameters is within the normal range, their combinations can result in abnormal gating.) Figure 4 illustrates the results of the 100 simulations.

The first question was how the observation that “FI_sb” can cause a shift of the “SInact_V” (“steady-state slow inactivation”) curve depends on model parameters. Out of the 100 simulations, 23 random sets of parameters did not result in substantial slow inactivation, so the shift of V_1/2_ could not be determined. In 50 out of the remaining 77 simulations, the shift was larger than −5 mV (Figure 4A). We plotted the values for the shift of inactivation curves against the difference between the V_1/2_ of the equilibrium curves for slow and fast inactivation (calculated from the randomly generated parameters of rate constants). Although a significant shift by “FI_sb” more frequently occurred when the *V_1/2_* for fast inactivation was more negative (i.e., in the right-hand side of the plot), in several cases it can be seen even at the other side. We can conclude that, in roughly two-thirds of the cases, fast-inactivated state-preferring drugs did shift the “SInact_V” curve. This suggests that a shift of the “steady-state slow inactivation” curve is certainly not a good indicator of slow-inactivated state preference.

To verify the second and third observations, the effect of all four prototypical drugs was tested in protocols “SInact_t” and “Rec_t.” The extent of the shift caused by individual drugs was quantified by calculating the sum of differences (SOD) between the curves in the control conditions and during drug application. Because the sampling points were evenly distributed on the logarithmic scale, the SOD was proportional to the area between the curves displayed on a semi logarithmic plot. For the sake of comparability, we calculated the normalized sum of differences (nSOD) by dividing SOD values by the sum of the control values (i.e., the full “area” under the control curve). The value of nSOD varied between 0 and 1; nSOD equaled 0 if the drug had no effect on slow inactivation or recovery, and equaled 1 if the current was completely abolished by the drug. Values of nSOD for the four prototypical drugs are shown in Figures 4B and C. For shifting the “SInact_t” curve, “FI_fb” was the most efficient drug for 90 out of 100 sets of parameters, while “FI_sb” and “SI_fb” were most efficient in 6 and 4 sets of parameters, respectively. The typical order of potency (for 55 out of 100 sets of parameters) was “FI_fb”>“FI_sb”>“SI_fb”>“SI_sb.” For shifting the “Rec_t” curve, “FI_sb” was most often (for 70 out of 100 sets) the most effective. The “SI_sb” and “SI_fb” were found to be most efficient in 20 and 8 sets, respectively. Note that, for both tests, one of the fast-inactivated state-preferring drugs typically performed better than either of the slow-inactivated preferring ones.

Summarizing the results of the Monte Carlo simulations, all three observations were verified for all models. We have demonstrated that “FI_sb” type drugs can behave like “SI” type drugs in “FInact_V” and “SInact_V” protocols. Therefore these protocols cannot be used for distinguishing “FI” and “SI” drugs. Nevertheless, we also observed definite tendencies, e.g., “FI” drugs tend to be more effective in “SInact_t” protocols than in “Rec_t” protocols, or “FI_sb” drugs tend to be the most effective in “Rec_t” protocols (Figure 3–4). We explored how much the combined information from the two protocols could tell us about SCI state preferences.

### Parameter-dependence of effectiveness in “SInact_t” and “Rec_t” protocols

**Figure 5:**
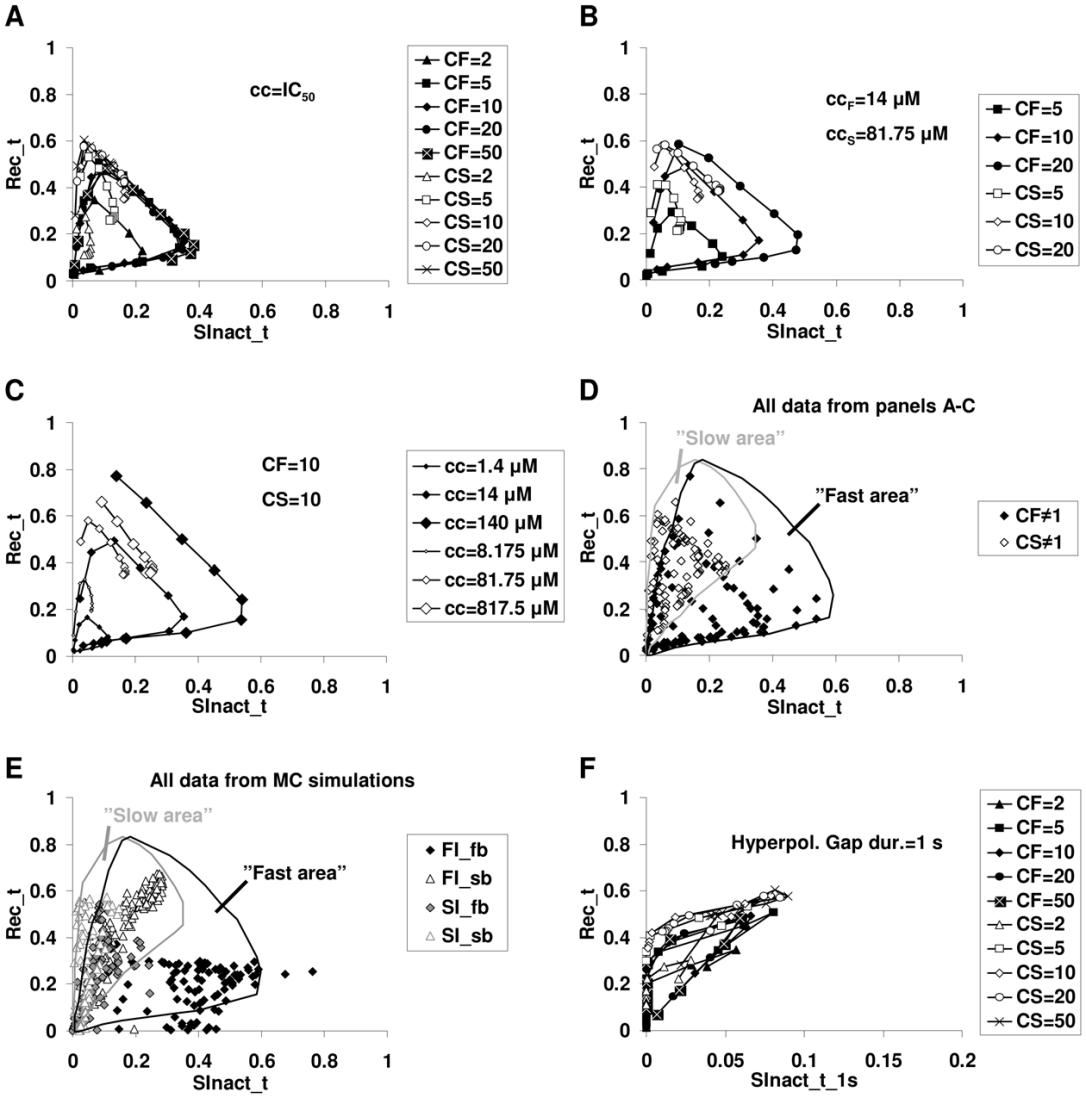
Different effectiveness of “FI” and “SI” drugs in two voltage protocols. Plots of effectiveness (quantified as nSOD, as described in text) in the “Rec_t” protocol plotted against effectiveness (nSOD) in the “SInact_t” protocol for various simulated drugs. Drugs with the same state preference factor (*CF* or *CS*) and concentration are connected. **A**, Distribution of 100 simulated drugs with different state preference factors and binding kinetics, applied in their IC_50_ (−90 mV) concentration. **B**, The effect of different state preference factors. Simulated drugs were applied in 14 µM concentrations in the case of fast inactivation preferring, and in 81.75 µM concentrations in the case of slow inactivation preferring drugs. **C**, The effect of different concentrations. The state preference factor was set to 10 in case of both fast and slow inactivation preferring drugs. **D**, All simulated drugs plotted on one figure. The areas of fast- and slow-inactivated preferring drugs were determined using all data points simulated. **E**, Prototypical drugs (“FI_fb,” “FI_sb,” “SI_fb,” and “SI_sb”) simulated using the 100 channel models from the Monte Carlo simulation. The two areas were defined based on the points shown in panel D. **F**, Effect of increasing the hyperpolarizing gap in the “SInact_t” protocol from 10 ms to 1 s. The 100 simulated drugs from panel A were applied in their IC_50_ (−90 mV) concentration.

We plotted the nSOD values of the “Rec_t” protocol as a function of the nSOD values of the “SInact_t” protocol. (Figure 5). We investigated the effect of changing the following parameters: i) binding kinetics of drugs, ii) state preference factors (*CF* and *CS*), iii) drug concentration, iv) sodium channel model parameters, and v) hyperpolarizing gap duration in the “SInact_t” protocol.

*Binding kinetics:* We simulated 10 different pairs of rate constants spanning five orders of magnitude from 5*10^−4^ to 15 µM^−1^s^−1^ (*k_a_*) and from 0.1 to 3000 s^−1^ (*k_d_*). The ratio of *k_a_* and *k_d_* was kept constant *k_a_/k_d_* = 5*10^−3^, ensuring that the affinity of the drug toward the resting channel remained constant.

*State preference factors*: *CF* and *CS* were given the following values: 2, 5, 10, 20 or 50. Using the five *CF* and the five *CS* values, each with the ten pairs of *k_a_* and *k_d_* values, we simulated altogether 100 “drugs” in both “SInact_t” and “Rec_t” protocols. To correct for different potencies, the concentration of each drug was scaled: we used the concentration that caused 50% inhibition of single depolarizations at −90 mV holding potential (Table S5). Figure 5A shows the distribution of “Rec_t” nSOD vs.“SInact_t” nSOD values. As the binding kinetics were accelerated, data points for specific *CF/CS* values proceeded clockwise along a closed loop. The explanation is that binding kinetics have a range of optimal effectiveness; kinetics that are too slow do not allow for sufficient association during depolarizations, while kinetics that are too fast cause drug molecules to dissociate more during hyperpolarizations. Around the optimum conditions, effectiveness in the “SInact_t” protocol increases with an acceleration in the kinetics in parallel with a decrease of effectiveness in the “Rec_t” protocol.

When *CF* and *CS* values were changed without concentration correction, the absolute value of the change was proportional to the value of *CF* and *CS*, but the characteristic clockwise loop pattern was unchanged (Figure 5B).

*Drug concentration*: The effect of changing concentrations while keeping *CF* or *CS* constant (*CF* = 10 or *CS* = 10) is shown in Figure 5C. The concentration was decreased and increased tenfold. The effect increased with increasing concentration, while acceleration of the binding kinetics caused the points to move along the clockwise loop as described above.

When all simulation results were plotted on the nSOD(Rec_t) – nSOD(SInact_t) plane, we observed that fast- and slow-inactivated state-stabilizing drugs were confined to limited but overlapping areas of the plane (Figure 5D). Because of the clockwise progression of the points upon acceleration of the binding kinetics, the overlapping area contains mostly “FI_sb” and “SI_fb” type drugs.

*Sodium channel model parameters*: To test the influence of channel parameters, we plotted the results from Monte Carlo simulations of the four prototypical drugs on the nSOD(Rec_t) – nSOD(SInact_t) plane, and compared those with the areas based on Figure 5D. “FI” drugs were almost exclusively located within the “fast area,” while “SI” drugs were located within the “slow area,” practically irrespective of model parameters. The overlapping area was populated mostly by “FI_sb” and SI_fb” drugs, confirming the reliability of “fast” and “slow” areas (Figure 5E).

*Hyperpolarizing gap duration of the “SInact_t” protocol*: In simulations and experiments, we used a 10 ms gap duration, which is enough for a >90% recovery from the fast-inactivated state under control conditions. In the presence of a fast-inactivated state-stabilizing drug, recovery is slowed down. For this reason, in experiments where slow-inactivated state-stabilizing drugs are to be identified, gap duration is often chosen to be of a longer duration (up to 1 s) to ensure that the recovery from fast inactivation is complete. Our simulations indicated that “FI_sb” and “SI” type drugs nevertheless overlap in behavior no matter what hyperpolarizing gap duration is chosen (see Figure 3E). We tested the effect of setting the gap duration to 1 s (Figure 5F). “FI” and “SI” type drugs were no better separated with a 1 s than with the 10 ms gap duration.

In summary, localization on the nSOD(Rec_t) – nSOD(SInact_t) plane can reveal the state preference of a drug if it falls on one of the non-overlapping areas. However, many “SI_fb” and “FI_sb” type drugs are expected to fall in the overlapping section and, therefore, their state preference cannot be determined.

### Electrophysiology experiments with SCIs

**Figure 6:**
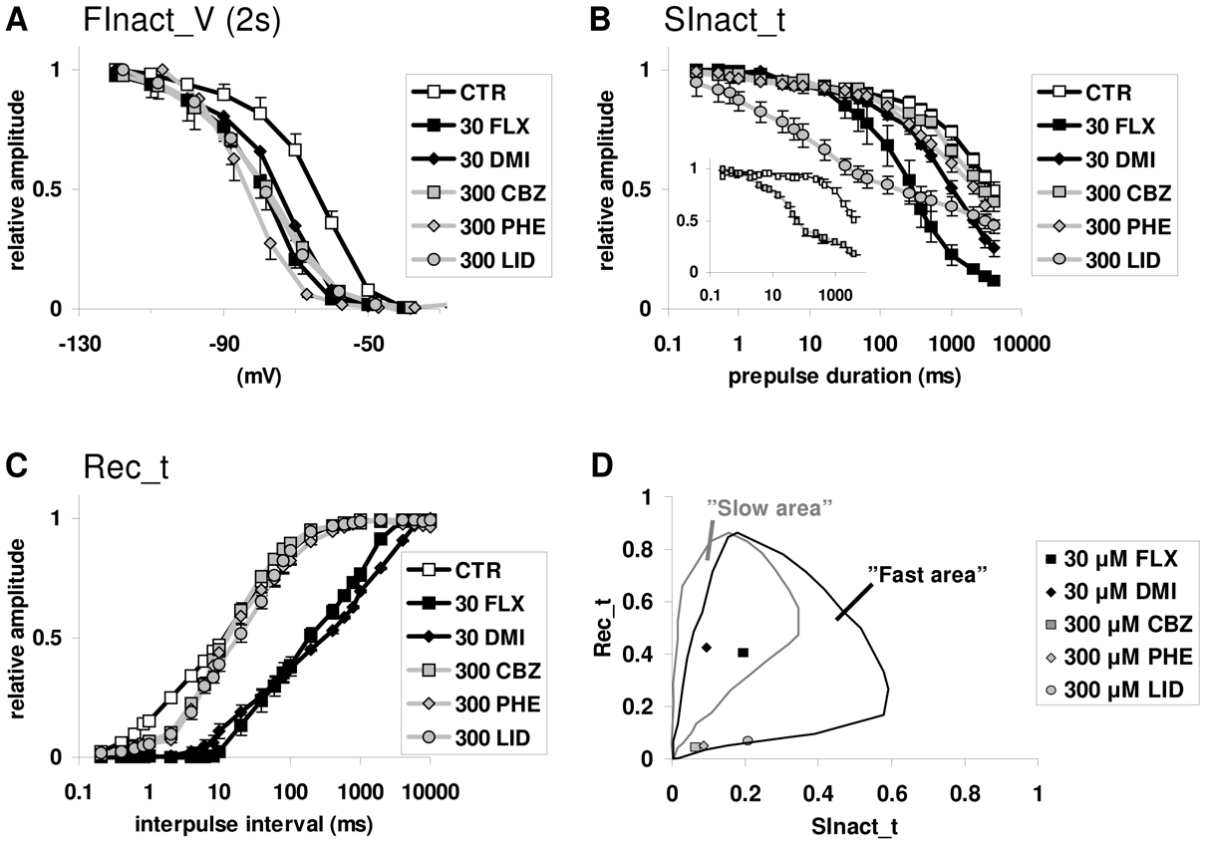
The effect of five SCIs in different voltage protocols. The following drugs were investigated: 30 µM fluoxetine (FLX, *black squares*), 30 µM desipramine (DMI, *black diamonds*), 300 µM carbamazepine (CBZ, *gray squares*), 300 µM phenytoin (PHE, *gray diamonds*) and 300 µM lidocaine (LID, *gray circles*). **A**, “FInact_V” protocol. Pre-pulse duration was set to 2 s. All drugs produced a similar voltage shift. **B**, “SInact_t” protocol. Carbamazepine and phenytoin induced only a minor modification compared to the control. Fluoxetine and desipramine produced a definite shift. Lidocaine caused inhibition even in the time range of fast inactivation. **C**, “Rec_t” protocol. Unlike fluoxetine and desipramine, carbamazepine, phenytoin and lidocaine only affected the first phase of the curve, which corresponds with recovery from the fast-inactivated state. **D**, The nSOD(Rec_t) – nSOD(SInact_t) plots for the drugs that were studied. Fluoxetine and desipramine occupied the overlapping area; plots of carbamazepine, phenytoin and lidocaine fell into the area of fast-inactivated state stabilization.

The following SCI drugs were used: the local anesthetic and antiarrhythmic lidocaine (300 µM), the anticonvulsants phenytoin (300 µM) and carbamazepine (300 µM), and the antidepressants fluoxetine (30 µM) and desipramine (30 µM). The concentrations were chosen to be similarly effective in causing a hyperpolarizing shift (−10 to −18 mV) of the “steady-state inactivation” curve (“FInact_V” – 2 s pre-pulse) (Figure 6A).

In the “SInact_t” protocol (Figure 6B), carbamazepine and phenytoin caused only a small acceleration in the process of inactivation. Fluoxetine and desipramine caused an obvious shift, similar to the one caused by the prototypical drugs “FI_sb,” “SI_fb” and “SI_sb.” Lidocaine strongly shifted the curve (especially in the early phase), which is typical of “FI_fb” type drugs. The reason for the small effect of carbamazepine was its fast dissociation kinetics. When the hyperpolarizing gap duration was changed from 10 ms at −150 mV to 5 ms at −120 mV (similar to the protocol used by Kuo et al. [12]), carbamazepine became as effective as lidocaine (Figure 6B inset).

In the “Rec_t” protocol (Figure 6C), fluoxetine and desipramine shifted the curve of recovery, similar to the prototypical drugs “FI_sb,” “SI_sb” and “SI_fb.” Carbamazepine, phenytoin and lidocaine only altered the early phase of the recovery curve, similar to the drug “FI_fb.”

We created the nSOD(Rec_t) – nSOD(SInact_t) plots for all five drugs (Figure 6D). The data points for fluoxetine and desipramine were in the overlapping area. The data points for carbamazepine, phenytoin and lidocaine fell into the non-overlapping area of fast inactivation stabilizing drugs.

## Discussion

Slow-inactivated state preference has been proposed to be a therapeutic advantage [19]–[21], and therefore different drugs have been tested for this property. The question of fast- or slow-inactivated state preference is a complex problem because of the interdependence of binding and gating equilibria. Multiple interconnected equilibria can be relatively easily handled by modeling; therefore, we used this approach to test hypotheses regarding state preference. Our current simulation data suggest that conclusions based on conventional protocols [19]–[21], [33]–[35] are not reliable.

A shift of the “steady-state slow inactivation curve” (“SInact_V” protocol), a shift of the “slow inactivation onset” curve (“SInact_t” protocol) and a shift of the recovery curve (“Rec_t” protocol) could all be caused by both fast- or slow-inactivated state stabilization. This conclusion was confirmed both by testing whether our observations were true for the entire parameter space and by applying a different type of model. We found that, with all combinations of parameters (within the reasonable range), our observations held true. Furthermore, both the phenomenological tetracube model and the MSA state model gave qualitatively similar results.

Nevertheless, the four prototypical mechanisms behaved appreciably differently. For this reason, we investigated the extent to which the two major mechanisms (“FI” and “SI”) could be distinguished using the combined information from different voltage protocols. Based on the nSOD(Rec_t) – nSOD(SInact_t) plots, we concluded that “FI” type drugs can be recognized, provided that their binding kinetics are fast enough. However, “FI” drugs with slower binding kinetics will overlap with “SI” drugs. Determination of the state preference would only be possible if we could measure the binding kinetics of individual drugs. However, distinguishing slow association from association to a slow-inactivated state is not trivial. In order to separate gating kinetics from binding kinetics, a rapid pulse application of the drug is necessary [32], [36]. Even in this case, association and dissociation rates cannot be correctly determined because the drug binding site on sodium channels is not extracellularly localized. Therefore, the onset rate of a drug effect may be determined by multiple processes: aqueous phase – membrane partitioning, outer to inner leaflet translocation, intramembrane diffusion and association, itself. Any one of these may be the rate limiting step, which obscures the microscopic association rate.

We investigated three well-known SCI drugs (lidocaine, phenytoin and carbamazepine) and two antidepressants (fluoxetine and desipramine). The uniquely high incidence of SCI activity among antidepressants [2], as well as their high affinity to sodium channels as compared to classic SCIs, suggests that the inhibition of sodium channels may contribute to their therapeutic effect. The therapeutic profile of antidepressants is different from that of classic SCIs (anticonvulsants, local anesthetics, antiarrhythmics), and we also intended to study whether the mechanism of inhibition was similar to that of classic SCIs.

The experimental behavior of the five drugs was remarkably similar to the behavior of prototypical drugs in simulations. We suggest that lidocaine, phenytoin and carbamazepine stabilize the fast-inactivated state, and that they have fast binding kinetics. Their nSOD(Rec_t) – nSOD(SInact_t) plot clearly fell into the “fast area.” Furthermore, their effect on the “Rec_t” curve was similar to the effect of “FI_fb.” Lidocaine behaved similarly to “FI_fb” in the “SInact_t” protocol as well. We hypothesized that the moderate effect of phenytoin and carbamazepine was due to their extra fast dissociation kinetics. This hypothesis was verified in the case of carbamazepine, which produced the characteristic “FI_fb” type effect on “SInact_t” curves upon minor modifications to the protocol.

The nSOD(Rec_t) – nSOD(SInact_t) plots of fluoxetine and desipramine fell into the overlapping area. Thus, their state preference could not be unambiguously determined. However, their properties of inhibition definitely differed from those of classic SCIs.

## Methods

### Electrophysiology

Patch clamp electrophysiology was done on native sodium channels in cultured hippocampal neurons. Cell culture preparation and electrophysiology were performed as published previously [32]. Cultured hippocampal neurons (prepared on the 17^th^ day after gestation) were found to express mostly the Nav1.2 and Nav1.6 isoform, but Nav1.1, Nav1.3 and Nav1.7 isoforms were also detected in a some cells [37]. In spite of the differences in expression pattern biophysical properties of sodium currents were remarkably similar [32], [37], and potency of individual drugs showed no higher variance than in experiments using Nav1.2 expressing HEK 239 cells (data not shown). Error bars on the figures represent SEM, and the number of cells tested (n) was between 4 to 10.

### Ethics statement

All experimental procedures were approved by the Animal Care and Experimentation Committee of the Institute of Experimental Medicine, and as stated by the decision of the Animal Health and Food Control Department of the Ministry of Agriculture and Regional Development, were in accordance with 86/609/EEC/2 Directives of European Community.

### Modeling

The simulation was based on a set of differential equations with the occupancy of each state (i.e., the fraction of the ion channel population in that specific state) given by the following equation:
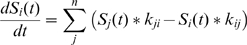
(1)where *S_i_(t)* is the occupancy of a specific state at time *t* and *S_j_(t)* is the occupancy of a neighboring state. Neighboring states are states where direct transitions are possible. *n* is the number of neighboring states, and *k_ij_* and *k_ji_* are the rate constants of transitions between neighboring states.

Differential equations were solved during simulations using a fourth-order Runge-Kutta method. We used either Berkeley Madonna v8.0.1 (http://www.berkeleymadonna.com/) or a program written in C++.

#### Description of the tetracube (Hodgkin-Huxley type) model

**Figure 7:**
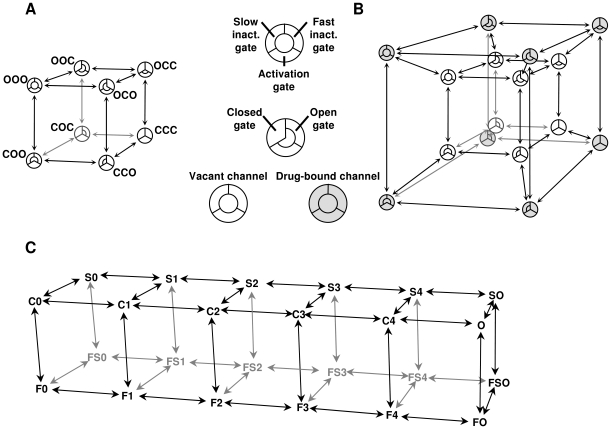
Topology of states in our models, as described in the text. **A**, Tetracube model of the drug-free channel. The position of the three gates is illustrated as a section of a circle. (This arrangement makes no reference to the structure of sodium channels.) **B**, Formation of the tetracube: Introduction of drug-bound states into the model. Drug association to all states was allowed. **C**, “Multi-step activation” (MSA) model of the drug-free channel.

The model in itself, not including drug effects, is equivalent to a modified Hodgkin-Huxley model of sodium channels; the gates were assumed to move independently but in a voltage-dependent manner. One modification is that, besides activation and fast inactivation gates, we included a slow inactivation gate. (The structural correlate of slow inactivation is thought to be a collapse of the outer pore region [38]. This mechanism may not be mechanistically called a gate, but for the sake of simplicity, we will define “gate” functionally, and consider any mechanism that can close and open the channel, a “gate”.) The model is phenomenological because we do not consider the interdependence between gating processes (all of our major findings were confirmed in a model where the processes of activation and inactivation were coupled – see Text S1). For this reason, individual states of the model do not necessarily correspond with actual conformations of the real channel. Channels were considered conducting when all three gates were open. The topology of the model can be illustrated by constructing a cube (Figure 7A), with the states forming the vertices and conformational transitions forming the edges. Open or closed conformations of the three gates (activation, fast inactivation and slow inactivation gates) define individual states of the channel and are shown by the three letters. The voltage-dependence of the rate constants is defined by an exponential equation:
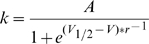
(2)where the three free parameters *A*, *V_1/2_* and *r* define the limiting value, the inflection point and the maximal slope on the log rate vs. voltage plot, respectively. The model thus far is no different from a Hodgkin-Huxley type model (which also supposes the independent movement of gates) except for the parameter “*m*” [39], which is not raised to the third power. The steepness of the activation vs. voltage plot can be achieved by choosing appropriate slope values for activation and deactivation, and the delay in the activation vs. time plot can be ignored. Although there is no difficulty in introducing multiple “*m*” particles in the state model, we decided to avoid this for the sake of simplicity. A detailed description of the model with numerical values of the 18 “ion channel-specific” parameters is shown in Text S1 and Table S3. These parameters reflect properties of the channel itself (not of the drug); therefore they were kept constant in all simulations in this study, except during the Monte Carlo study when the effect of the “ion channel-specific” parameters was investigated. “Drug-specific” parameters are described in the following section.

#### “Drug-specific” parameters: Introduction of drug effects into the tetracube model

Because we supposed that drug association to all states is possible, we connected a drug-bound state to each vacant state, thus forming the tetracube (tesseract) topology of the model (Figure 7B). Two additional (voltage-independent) rate constants were defined: *k_a_* (association rate constant; units: s^−1^µM^−1^) and *k_d_* (dissociation rate constant; units: s^−1^). These were defined to determine the affinity of the drug to the resting state. Drug-induced changes in gating rates were simulated as predicted by the “modulated receptor hypothesis” [26], [27], i.e., drug association to all states was made possible but not with equal affinity. The model also incorporates one major assumption of the “guarded receptor hypothesis” [24] in the sense that besides increased affinity to the relevant inactivated state, accessibility of the binding site (determining association and dissociation rates) was also increased. The same three gate-specific factors (*CA*, *CF* and *CS*) were used to determine both differences in affinity (association-dissociation equilibria) and differences in the relevant gating equilibria (for a detailed description of the model see Text S1). Using this constraint, microscopic reversibility was maintained in the model. Three constants were defined to correspond to each of the three gates: “Closed Activation gate stabilizing factor” (*CA*), “Closed Fast inactivation gate stabilizing factor” (*CF*) and “Closed Slow inactivation gate stabilizing factor” (*CS*). Values larger than 1 were defined as being able to the closed conformation of the gate, while values less than 1 stabilized the open conformation. The calculation of individual rate constants is described in Table S1 and Table S2.

In order to describe the specific drug effects on the channels, we have defined five “drug-specific” parameters: *k_a_*, *k_d_*, *CA*, *CF*, and *CS*. We assumed that drug-bound channels are blocked, i.e., they are not able to conduct. This may not be true for all SCIs, but in order to be able to explain inhibition at strongly hyperpolarizing holding potentials (e.g. −150 mV), this was the simplest hypothesis to assume. Also, several SCIs have been shown to affect open channels preferentially. In this study our aim was to follow the dynamics of resting, fast inactivated and slow inactivated states in the absence and presence of drugs, therefore we did not address this mechanism, thus *CA* equaled 1 in all simulations.

While the affinity of the drug to resting channels is determined by the *k_a_*/*k_d_* ratio, the apparent affinity to the whole channel population, measured in a specific protocol must differ from this value and is determined by all channel-specific and drug-specific parameters, as well as by the parameters of the test protocol.

#### Description of the multi-step activation (MSA) model

Although the tetracube model reproduces the voltage-dependent kinetics of major gating transitions (activation, deactivation, fast and slow inactivation, as well as recovery from both inactivated states) fairly well, it obviously oversimplifies the gating mechanisms. Most notably, unlike in real sodium channels, activation is a single-step process, both types of inactivation are voltage-dependent in themselves, and the gating transitions are independent. A model in which activation is a multi-step process with several intermediate states could better reproduce channel behavior. In addition, using such a model, both fast and slow inactivation themselves can be made voltage-independent (deriving their voltage-dependence from the movement of voltage sensors), which is a more correct approximation of real channel behavior. Furthermore, using this type of model would enable researchers to test alternative state preferences, such as the preference for intermediate states [14], [15], [40]. This type of model has been used previously in different studies [15], [31], [41], but none of these models included slow inactivation. In order to verify our conclusions obtained with the tetracube model, we built an MSA model with both fast- and slow-inactivated states.

We used the model described by Kuo and Bean [31] as a starting point. We used three-parameter exponential equations (Eq. #2) to describe the voltage-dependence of the rate constants, instead of the original two-parameter equations, because the latter have no maxima, and therefore simulations tend to be unstable at extreme membrane potential values.

In the model (Figure 7C), horizontal transitions (except the rightmost one) correspond to the voltage-dependent movement of voltage sensors, vertical transitions correspond to the movement of the fast-inactivation particle, and backward-forward transitions correspond to the movement of the slow inactivation gate. This model, too, is considerably simplified: it assumes that voltage sensors are identical and independent, that opening requires all four voltage sensors to be in the depolarized positions and that inactivation depends equally on the positions of the four voltage-sensors. A detailed description of the states and calculation of transitions are in Text S1, Table S1 and Table S2; parameters of the model, and constraints for optimization are in Table S4.

Reproductions of the currents, “steady-state inactivation” (“FInact_V”) and activation curves, as well as the “SInact_t” and “Rec_t” curves by both the tetracube and MSA models, are shown in Figure S1. The MSA model was superior to the tetracube model in the accuracy of the activation kinetics, but the price for this is paid in a radically increased computational demand (48 states and 224 transitions instead of 16 states and 64 transitions).

#### Introduction of drug effects into the MSA model

We added drug-bound states, following the same principle as described for the tetracube model (see Figure 7B). We doubled the number of states (adding a drug-bound state for each unbound state), and used the same factors (such as *CF* or *CS*) to calculate the differences both in state-dependent affinity and in drug-binding-site-occupancy-dependent gating equilibria (see Text S1, Table S1 and Table S2). (Note that, by this method, Markov-type models of any complexity can be made to handle modulated receptor hypothesis-based drug effects, while detailed balance in the model is maintained.)

#### Monte Carlo simulations

In order to test the robustness of our findings, we performed simulations with the tetracube model using random parameters. The program was written in C++. The constraints used for random number generation are given in Table S3. Uniformly distributed random numbers were generated between 0 and 1 and then transformed either linearly (in the case of *V_1/2_* values – see Eq. #2) or logarithmically (in the case of *A* and *r* values – see Eq. #2) to match the limits shown in Table S3.

## Supporting Information

Figure S1 Evaluation of the goodness of fit during optimization of the models
(0.39 MB TIF)

Figure S2 Results of simulations with the MSA model using the four prototypical mechanisms
(0.78 MB TIF)

Table S1 The effect of drug binding on rate constants
(0.03 MB DOC)

Table S2 Calculation of association and dissociation rate constants
(0.03 MB DOC)

Table S3 Ion channel-specific parameters of the tetracube model
(0.03 MB DOC)

Table S4 Ion channel-specific parameters of the MSA model
(0.03 MB DOC)

Table S5 IC50 values for simulated drugs with different CF or CS factors
(0.03 MB DOC)

Text S1 Detailed description of the “tetracube” and “MSA” models
(0.09 MB DOC)
