# Supplementary figures and images for: Fast- or Slow-inactivated State Preference of Na+ Channel Inhibitors: A Simulation and Experimental Study
Source: PLoS Comput Biol. 2010 Jun 17;6(6):e1000818. doi: 10.1371/journal.pcbi.1000818 (PMC2887460; doi:10.1371/journal.pcbi.1000818)

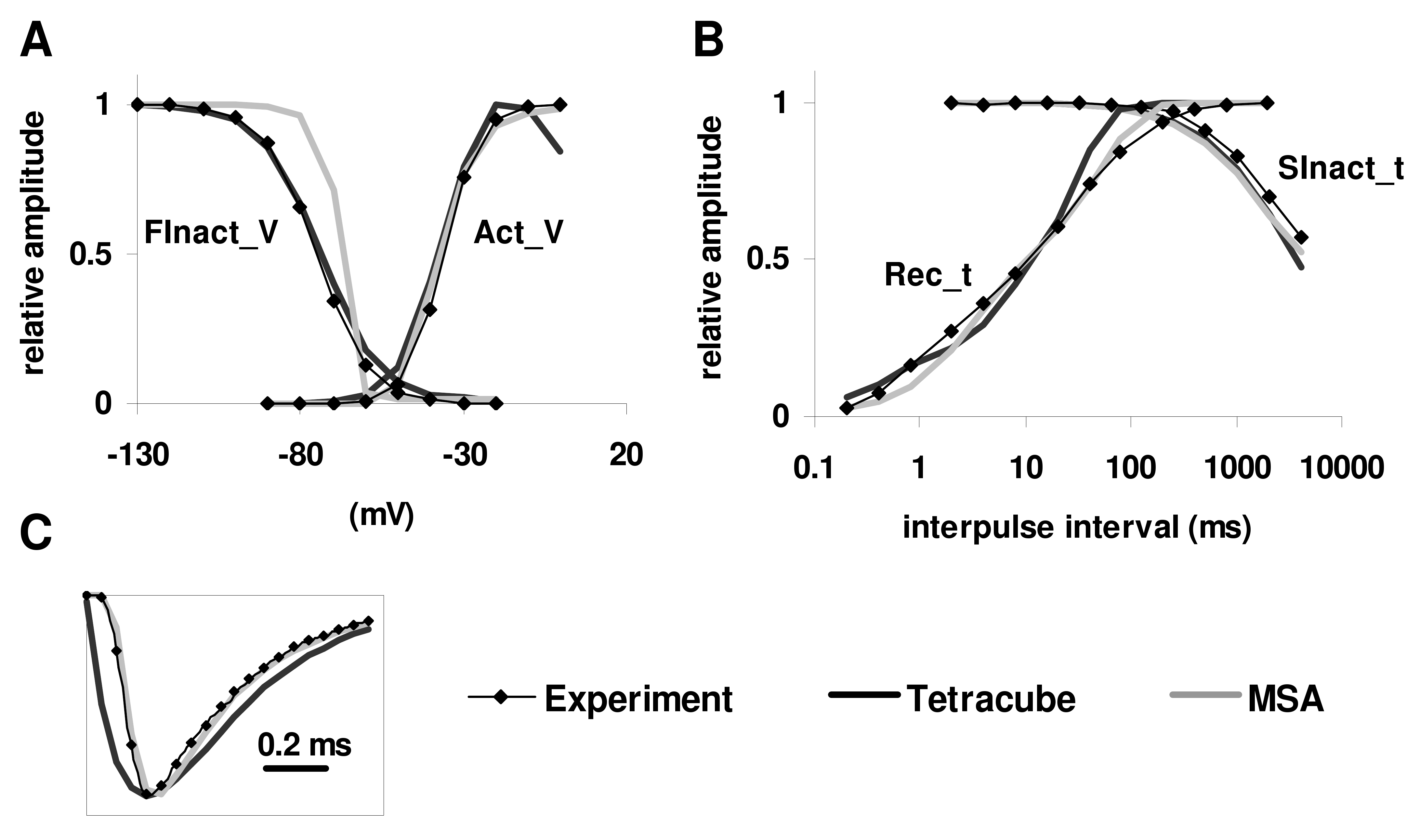

Supplement: Figure S1 — Evaluation of the goodness of fit during optimization of the models (0.39 MB TIF) [file pcbi.1000818.s001.tif]

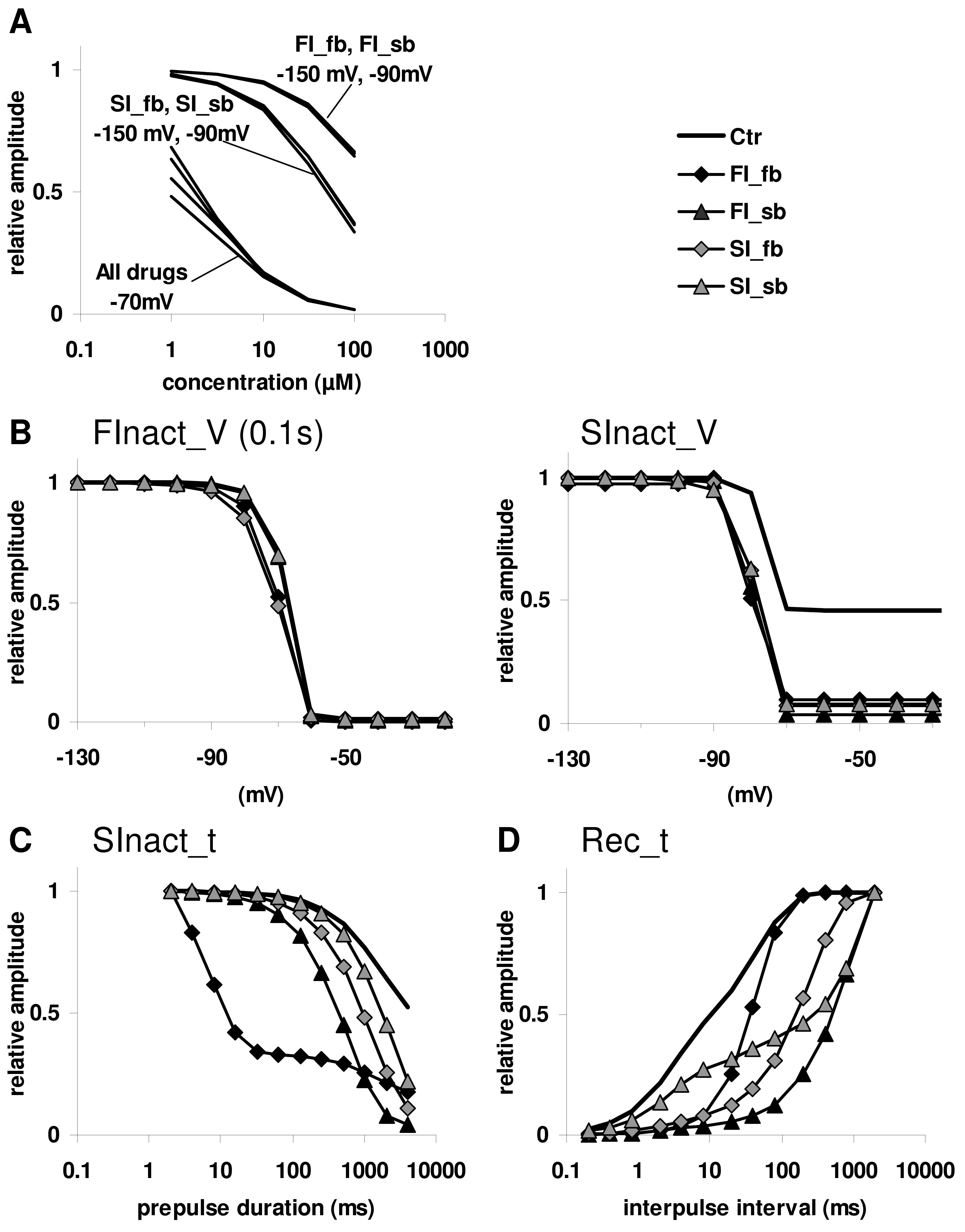

Supplement: Figure S2 — Results of simulations with the MSA model using the four prototypical mechanisms (0.78 MB TIF) [file pcbi.1000818.s002.tif]
