# Supplementary material for: Fast- or Slow-inactivated State Preference of Na+ Channel Inhibitors: A Simulation and Experimental Study
Source: PLoS Comput Biol. 2010 Jun 17;6(6):e1000818. doi: 10.1371/journal.pcbi.1000818 (PMC2887460; doi:10.1371/journal.pcbi.1000818)
Supplement: Table S1 — The effect of drug binding on rate constants (0.03 MB DOC) [file pcbi.1000818.s003.doc]

Tetracube model:

| **Unbound** | **Bound** |
| --- | --- |
| k_aO_ | k_aO_/CA |
| k_aC_ | k_aC_*CA |
| k_fO_ | k_fO_/CF |
| k_fC_ | k_fC_*CF |
| k_sO_ | k_sO_/CS |
| k_sC_ | k_sC_*CS |

MSA model:

| **Unbound** | **Bound** |
| --- | --- |
| fic_n_ | fic_n_*CF |
| fio | fio*CF |
| frc_n_ | frc_n_/CF |
| fro | fro/CF |
| sic_n_ | sic_n_*CS |
| sio | sio*CS |
| sic_n_ | sic_n_/CS |
| sro | sro/CS |
