# Supplementary material for: Fast- or Slow-inactivated State Preference of Na+ Channel Inhibitors: A Simulation and Experimental Study
Source: PLoS Comput Biol. 2010 Jun 17;6(6):e1000818. doi: 10.1371/journal.pcbi.1000818 (PMC2887460; doi:10.1371/journal.pcbi.1000818)
Supplement: Table S2 — Calculation of association and dissociation rate constants (0.03 MB DOC) [file pcbi.1000818.s004.doc]

Tetracube model:

|  | **Association** | **Dissociation** |
| --- | --- | --- |
| **OOO** | k_a_*cc/CA | k_d_*CA |
| **COO** | k_a_*cc | k_d_ |
| **OCO** | k_a_*cc*CF/CA | k_d_*CA/CF |
| **OOC** | k_a_*cc*CS/CA | k_d_*CA/CS |
| **OCC** | k_a_*cc*CF*CS/CA | k_d_*CA/(CF*CS) |
| **COC** | k_a_*cc*CS | k_d_/CS |
| **CCO** | k_a_*cc*CF | k_d_/CF |
| **CCC** | k_a_*cc*CF*CS | k_d_/(CF*CS) |

MSA model:

|  | **Association** | **Dissociation** |
| --- | --- | --- |
| **C_n_ or O** | k_a_*cc | k_d_ |
| **F_n_ or FO** | k_a_*cc *CF*cc | k_d_/CF |
| **S_n_ or SO** | k_a_*cc *CS | k_d_/CS |
| **FS_n_ or FSO** | k_a_*cc *CF*CS | k_d_/(CF*CS) |
