# Supplementary material for: Fast- or Slow-inactivated State Preference of Na+ Channel Inhibitors: A Simulation and Experimental Study
Source: PLoS Comput Biol. 2010 Jun 17;6(6):e1000818. doi: 10.1371/journal.pcbi.1000818 (PMC2887460; doi:10.1371/journal.pcbi.1000818)
Supplement: Table S3 — Ion channel-specific parameters of the tetracube model (0.03 MB DOC) [file pcbi.1000818.s005.doc]

|  | **Activation gate Opening** | **Activation gate Closing** | **Fast Inactivation gate Opening** | **Fast Inactivation gate Closing** | **Slow Inactivation gate Opening** | **Slow Inactivation gate Closing** |
| --- | --- | --- | --- | --- | --- | --- |
| **A (ms^-1^)** | 4.581  (1; 10) | 8.337  (2; 20) | 2.351  (0.5; 3) | 24.627  (1; 50) | 0.03998  (0.0005; 0.2) | 0.001084  (1e-4; 5e-3) |
| **V_1/2_ (mV)** | -20.016  (-50; -20) | -59.8799  (-90; -300) | -122.752  (-200; -45) | 38.892  (-50; 80) | -97.343  (-200; -45) | 65.0632  (-50; 80) |
| **r (mV)** | 8.836  (1; 20) | -9.6116  (-20; -1) | -15.726  (-20; -5) | 20.131  (5; 30) | -9.438  (-30; -5) | 98.72  (5; 100) |
