# Supplementary material for: Fast- or Slow-inactivated State Preference of Na+ Channel Inhibitors: A Simulation and Experimental Study
Source: PLoS Comput Biol. 2010 Jun 17;6(6):e1000818. doi: 10.1371/journal.pcbi.1000818 (PMC2887460; doi:10.1371/journal.pcbi.1000818)
Supplement: Table S4 — Ion channel-specific parameters of the MSA model (0.03 MB DOC) [file pcbi.1000818.s006.doc]

|  | **α** | **β** | **γ** | **δ** |
| --- | --- | --- | --- | --- |
| **A (ms^-1^)** | 35.925  (10; 100) | 200  (50; 500) | 31.765  (10; 100) | 73.182  (10; 100) |
| **V_1/2_ (mV)** | -36.203  (-60; 0) | -108.383  (-180; -80) | -28.216  (-90; 0) | -125.614  (-170; -70) |
| **r (mV)** | 15.339  (1; 30) | -14.012  (-30; -1) | 5.244  (1; 50) | -3.21255  (-20; -1) |

|  | **Fast Inactivation gate Opening** | **Fast Inactivation gate Closing** | **Slow Inactivation gate Opening** | **Slow Inactivation gate Closing** |
| --- | --- | --- | --- | --- |
| **C_0_, F_0_, S_0_, FS_0_ (ms^-1^)** | 0.4  (0.01; 15) | 0.0000374  (1e-5; 1e-2) | 0.021861  (0.001; 0.1) | 0.000562  (1e-5; 1e-2) |
| **O, OF, OS, OFS (ms^-1^)** | 0.04  (0.002; 0.2) | 3.421  (1; 15) | 0.000214  (1e-4; 1e-1) | 0.0004  (2e-5; 2e-3) |
