# Supplementary material for: Fast- or Slow-inactivated State Preference of Na+ Channel Inhibitors: A Simulation and Experimental Study
Source: PLoS Comput Biol. 2010 Jun 17;6(6):e1000818. doi: 10.1371/journal.pcbi.1000818 (PMC2887460; doi:10.1371/journal.pcbi.1000818)
Supplement: Table S5 — IC50 values for simulated drugs with different CF or CS factors (0.03 MB DOC) [file pcbi.1000818.s007.doc]

| **CF/CS** | **CF, IC_50_ (μM)** | **CS, IC_50_ (μM)** |
| --- | --- | --- |
| **2** | 142 | 191.6 |
| **5** | 47 | 148 |
| **10** | 14 | 81.75 |
| **20** | 3.6 | 29.3 |
| **50** | 0.6 | 5.3 |
