# Supplementary material for: Fast- or Slow-inactivated State Preference of Na+ Channel Inhibitors: A Simulation and Experimental Study
Source: PLoS Comput Biol. 2010 Jun 17;6(6):e1000818. doi: 10.1371/journal.pcbi.1000818 (PMC2887460; doi:10.1371/journal.pcbi.1000818)
Supplement: Text S1 — Detailed description of the “tetracube” and “MSA” models (0.09 MB DOC) [file pcbi.1000818.s008.doc]

**Detailed model descriptions**

**Circular models: General introduction** If we take a simple model where there are two states resting, (*R*) and inactivated (*I*), and the drug is able to bind to both states, the process can be modeled as follows:

Rate constants of association and dissociation are denoted *k_a_* and *k_d_*, respectively, voltage-dependent rate constants of gating are denoted *α* and *β*. The affinity of *R* and *I* states is supposed to be unequal, *A* and *D* represent the changes in association and dissociation rate constants for *I* state as compared to *R* state. *X* and *Y* are the changes in gating rates caused by drug binding. In the general case *A*, *D*, *X* and *Y* can take any value, if there is no change their value is 1, if there is an increase their value is greater than 1, and in case of a decrease their value is smaller than 1. Because this is a circular model the principle of detailed balance applies, i.e., the product of the rate constants clockwise must equal the product of the rate constants in the counterclockwise direction.

$$\begin{matrix} \alpha*k_{a}*A*\beta*Y*k_{d}=k_{a}*\alpha*X*k_{d}*D*\beta\\ A*Y=X*D \\ \frac{A}{D}=\frac{X}{Y} \end{matrix}$$

For this reason not all four parameters are independent, after choosing three the forth one has to be computed to satisfy the constraint. We can break up the four parameters into three independent parameters the following way:

$$\begin{matrix} A=C*Z \\ D=\frac{Z}{C} \\ X=C*Q \\ Y=\frac{Q}{C} \end{matrix}$$

This way the constraint of detailed balance will be satisfied by any value of *C*, *Z* and *Q*. (This is only one of the many possible ways of breaking up the four parameters into three independent ones.)

*C* will determine the rate of affinity between *R* and *I* states, as well as the rate of gating equilibria between unbound and bound channels. This means that it gives the free energy level of *BI* state, while *Z* and *Q* will modify the height of the energy barrier in the *I ↔ BI* and *BR ↔ BI* transitions, respectively. (If *R* state is defined as zero level, the level of *I* is determined by the *β/α* ratio, and the level of *BR* is determined by the *k_d_/ k_a_* ratio).

In the manuscript for the sake of simplicity we present data where *Z* = *Q* = 1. We performed simulations with *Z* ≠ 1, but in the protocols we used, the change of *Z* value caused only minor changes, and did not affect our main conclusions. We used the same “*C*” factor for modifying both onward and backward rates, because this way we could model a certain change in affinity with the least change in transition rates. This condition also means that both affinity and accessibility of inactivated state is increased, thus the model involves the main assumptions of both the modulated receptor hypothesis and the guarded receptor hypothesis.

**Detailed description of the “tetracube” (Hodgkin-Huxley type) model** The model in itself, not including drug effects, is equivalent to a modified Hodgkin-Huxley model of sodium channels, in which the probability of open channels is given by the product of the probabilities of each gate being in a permissive state. One modification is that, besides activation and fast inactivation gates, we included a slow inactivation gate. The other significant modification is that we wanted to include was drug effects. We supposed that drug association is possible for every combination of the gates being closed or open. Therefore, in order to construct a model in which this would be possible, we created a phenomenological state model. With such a model, simulation of drug association to and dissociation from different states, along with simulation of altered rate constants, can be readily performed. The model is phenomenological because we do not consider the interdependence between gating processes. In reality, the voltage-dependence for both inactivation processes comes in large part from the activation process; they are not strongly voltage-dependent by themselves (this condition is realized in our other model, described below). For this reason, individual states of the model do not necessarily correspond with actual conformations of the real channel. Channels were considered conducting when all three gates were open (as seen in the paper, Figure 6A; the schematic representation is not intended to make any reference to the structure of sodium channels). For simplicity, the gates were assumed to move independently, but each in a voltage-dependent manner. (All our major findings were confirmed in a model in which the processes of activation and inactivation were coupled – see below.) The topology of the model can be illustrated by constructing a cube (paper, Figure 6A), with the states forming the vertices and conformational transitions taking place along the edges. Open or closed conformations of the three gates (activation, fast inactivation and slow inactivation gates) define individual states of the channel, and are shown by the three letters. Starting from the open conformation (all three gates are open; denoted as “OOO”), three gating transitions can occur: deactivation (⇒ COO), fast inactivation (⇒ OCO) and slow inactivation (⇒ OOC). In the same way, all eight possible combinations of gate positions (closed or open) are defined as separate conformational states of the channel, and can be connected with neighboring states via three different conformational transitions. States at which the fast but not the slow inactivation gate is closed (OCO and CCO) can be collectively named fast-inactivated, while all states with the slow inactivation gate closed are considered slow-inactivated (OOC, OCC, COC and CCC). The resting state is when only the activation gate is closed (COO). Because gates, were defined to move independently, all parallel opening or closing transitions have identical but voltage-dependent rate constants. The voltage-dependence of the rate constants is defined by an exponential equation:

$k_{xY}=\frac{A}{1+e^{(V_{1/2}-V)*r^{-1}}}$ (1)

where “x” indicates the identity of the gates (it can be either a, f, and s, for activation, fast inactivation and slow inactivation gates, respectively) and “Y” indicates the direction of the transition (either C for closing, or O for opening). Thus, for example, the channel opening rate is denoted “k_aO._” Notations for the transition rates are shown in Table S1 (upper panel). The three free parameters *A*, *V_1/2_* and *r* define the limiting value, the inflection point and the maximal slope on the log rate vs. voltage plot, respectively. The model thus far is no different from a Hodgkin-Huxley type model (which also supposes the independent movement of gates) except for the parameter “m” [1], which is not raised to the third power. The steepness of the activation vs. voltage plot can be achieved by choosing appropriate slope values for activation and deactivation, and the delay in the activation vs. time plot can be ignored. Although there is no difficulty in introducing multiple “m” particles in the state model, for the sake of simplicity we decided to avoid this. Microscopic reversibility was imposed on the model by defining parallel rate constants (i.e., constants defining transition rates along parallel edges of the cube) as being identical. The full (drug-free) sodium channel model thus can be constructed by defining six voltage-dependent rate constants (that is 18 free parameters). Because these parameters are “ion channel-specific,” i.e., properties of the channel itself (not of the drug), they were kept constant in all the simulations comprising this study except during the Monte Carlo study when the effect of the “ion channel-specific” parameters was investigated. “Drug-specific” parameters are described in the following section.

**“Drug-specific” parameters: Introduction of drug effects into the tetracube model** Because we supposed that drug association to all states was possible, we connected a drug-bound state to each vacant state, thus forming the tetracube (tesseract) topology of the model (paper, Figure 6B). Two additional (voltage-independent) rate constants were defined: *k_a_* (association rate constant; units: s^-1^μM^-1^) and *k_d_* (dissociation rate constant; units: s^-1^). These were defined to determine the affinity of the drug to the resting state. Drug-induced changes in gating rates were simulated as predicted by the modulated receptor hypothesis, i.e., drug association to all states was made possible, but not with equal affinity.

The same three gate-specific factors (*CA*, *CF* and *CS*) were used to determine both differences in affinity (association-dissociation equilibria) and differences in the relevant gating equilibria. Using this constraint, microscopic reversibility was maintained in the model. The transition rates of drug-bound channels were modified by multiplying the closing rate of one of the gates by a constant and dividing the opening rate by the same constant. Three constants were defined to correspond to each of the three gates: “Closed Activation gate stabilizing factor” (*CA*), “Closed Fast inactivation gate stabilizing factor” (*CF*) and “Closed Slow inactivation gate stabilizing factor” (*CS*). Values larger than 1 were defined as being able to stabilize the closed conformation of the gate, while values less than 1 stabilized the open conformation. With *CS* = 2, for example, the rate of slow inactivation is doubled, while the rate of recovery from slow inactivation is halved at all voltages. In order to maintain detailed balance, binding rates were also modified with the same factors. In the above example, if *CS* = 2, association rates to slow-inactivated states were doubled, while dissociation rates were halved. While each individual gating rate of drug-bound channels is multiplied or divided by only one of the constants, individual dissociation rates can be multiplied or divided by one (OOO, COC, and CCO), two (OOC, OCO, and CCC), three (OCC), or zero (in the case of the resting state, COO) constants. (For example, association and dissociation rates for the resting channel are given by *k_a_* and *k_d_*, respectively, while association and dissociation rates for the slow-inactivated channel in the “OCC” conformation are given by *k_a_***CF***CS***CA*^-1^ and *k_d_***CF*^-1^**CS*^-1^**CA*, respectively.) Calculations for gating rate constants for drug-bound channels are given in Table S1 (upper panel), while association and dissociation rate constants from the model are shown in Table S2 (upper panel).

In order to describe specific drug effects on the channels, we have therefore defined five “drug-specific” parameters: *k_a_*, *k_d_*, *CA*, *CF*, and *CS*. We assumed that drug-bound channels are blocked, i.e., they are not able to conduct. This may not be true for all SCIs, but in order to explain inhibition at strongly hyperpolarizing holding potentials (e.g. -150 mV), this simple hypothesis must be assumed.

While the affinity of the drug to resting channels is determined by the *k_a_* / *k_d_* ratio, the apparent affinity, seen in a specific protocol, to the whole channel population must differ from this value, and is determined by all channel-specific and drug-specific parameters, as well as by the parameters of the test protocol.

**Description of the multi-step activation (MSA) model** Although the tetracube model reproduces the voltage-dependent kinetics of major gating transitions (activation, deactivation, fast and slow inactivation, as well as recovery from both inactivated states) fairly well, it obviously oversimplifies the gating mechanisms. Most importantly, unlike the real sodium channels, activation is a single-step process, both types of inactivation are voltage-dependent in themselves, and the gating transitions are independent. A model in which activation is a multi-step process with several intermediate states could better reproduce channel behavior. In addition, using such a model, both fast and slow inactivation can be made voltage-independent (deriving their voltage-dependence from the movement of voltage sensors), which is a more correct approximation of real channel behavior. Furthermore, using this type of model would enable researchers to test alternative state preferences, such as the preference for intermediate states ([2-4]). This type of model has been used previously in different studies [2,5,6], but none of these models included slow inactivation. In order to verify our conclusions, obtained with the tetracube model, we built an MSA model with both fast- and slow-inactivated states.

We used the model described by Kuo and Bean [6] as a starting point. We used three-parameter exponential equations (Equation #1) to describe the voltage-dependence of the rate constants, instead of the original two-parameter equations, because the latter have no maxima, and therefore simulations tend to be unstable at extreme membrane potential values.

In the model (paper, Figure 6C) horizontal transitions (except the rightmost one) correspond to voltage-dependent movements of the voltage sensors, vertical transitions correspond to the movement of fast inactivation particles, and backward-forward transitions correspond to the movement of the slow inactivation gate. This model, too, is considerably simplified: it assumes that voltage sensors are identical and independent, that opening requires all four voltage sensors to be in the depolarized positions and that inactivation depends equally on the positions of the four voltage-sensors. All these assumptions are known to be wrong. However, as we have said before, a correct model of sodium channels would require too many unknown independent parameters to be constructed unambiguously, and for our current purpose a simplified model serves well enough.

Individual states are denoted as C_n_ for closed, F_n_ for fast-inactivated, S_n_ for slow-inactivated and FS_n_ for the states at which both inactivated gates are closed, where n indicates the number of voltage sensors at the outward position. Rate constants for individual transitions are denoted as follows:

C_n-1_ → C_n_ = *α_n_*

C_n_ → C_n-1_ = *β _n_*

F_n-1_ → F_n_ = *αf_n_*

F_n_ → F_n-1_ = *βf_n_*

S_n-1_ → S_n_ = *αs_n_*

S_n_ → S_n-1_ = *βs_n_*

FS_n-1_ → FS_n_ = *αfs_n_*

FS_n_ → FS_n-1_ = *βfs_n_*

C_4_ → O = F_4_ → FO = S_4_ → SO = FS_4_ → FSO = *γ*

O → C_4_ = FO →F_4_ = SO → S_4_ = FSO → FS_4_ = *δ*

The voltage-dependence of the transition rates is given by Equation #1.

Rates of fast inactivation and recovery for unbound channels are denoted as ‘fic_n_’ and ‘frc_n_’ for the closed states, and ‘fio’ and ‘fro’ for the open states. The corresponding rates for slow inactivation and recovery are ‘sic_n_,’ ‘src_n_,’ ‘sio’ and ‘sro.’ Individual rates are calculated as follows:

fic_n_ = fic_0_*a^n^

frc_n_ = frc_0_/a^n^

sic_n_ = sic_0_*b^n^

src_n_ = src_0_/b^n^

where ‘a’ and ‘b’ are allosteric factors, calculated as follows:

a = (( frc_0_ / fic_0_)/( fro / fio)) ^1/8^

b = (( src_0_ / sic_0_)/( sro / sio)) ^1/8^

To ensure detailed balance, and to minimize the number of free parameters, voltage-dependent transition rates are derived from the transition rates of closed states, in the following way:

αf_n_ = α_n_ * a

βf_n_ = β_n_ / a

αs_n_ = α_n_ * b

βs_n_ = β_n_ / b

αfs_n_ = α_n_ * a * b

βfs_n_ = β _n_ / (a * b)

**Introduction of drug effects into the MSA model** We added drug-bound states, following the same principle as described for the tetracube model (see paper, Figure 6B): we doubled the number of states (adding a drug-bound state for each unbound state), and used the same factors (such as CF or CS) to calculate differences both in the state-dependent affinity (Table S1) and in the drug-binding-site-occupancy-dependent gating equilibria (Table S2). (Note that by this method, Markov-type models of any complexity can be made to handle modulated receptor hypothesis-based drug effects, while detailed balance in the model is maintained.)

**Parameter optimization**

**Optimization of “ion channel-specific” parameters** Parameter optimization was based on either our experimental data or on published analyses of sodium channel kinetics [7,8]. As one would expect, data obtained using different preparations and different experimental protocols differ somewhat, but our aim in this study was not to faithfully reproduce all kinetic features of the channel; we wanted to address the particular question of fast- vs. slow-inactivated state preference. All conclusions drawn in this paper were tested for robustness, as described below.

We optimized the parameters iteratively by combining manual adjustment with an optimization algorithm, to avoid local minima.

**Manual adjustment of parameters:** For the tetracube model, two sets of data can be determined experimentally for all three gates: steady-state equilibrium values and time constants of equilibration. The steady-state equilibrium distribution of channels, with the particular gate being open vs. closed as a function of voltage, was determined by standard activation and steady-state inactivation protocols; the pre-pulse duration of the latter was varied. The time constant of equilibration as a function of voltage was studied using the following protocols: time constant of activation and deactivation as a function of voltage was studied previously [9-11], and the voltage dependence of these data matched well with our experimental data on activation and deactivation. Time constants of fast inactivation could be estimated from the decay time constants of currents if the rate and delay of activation at different voltages was known. Because our model disregards the delay in activation, we set inactivation rate constants somewhat higher so that simulated currents could reproduce the experimentally observed kinetics. Time constants of recovery from fast inactivation were measured at two membrane potential values (-70 and -150 mV) using a double pulse protocol [12]. The voltage-dependence of slow inactivation and recovery from slow inactivation were studied using the “SInact_t” and “Rec_t” protocols (paper, Figure 1C-D). The voltage-dependent rate constants of opening “k_O_” and closing “k_C_” for each gate were determined from equilibrium-voltage and time constant-voltage plots (as described by O’Leary [13]), using the following equations: the equilibrium occupancy of an open state (E_O_) is given by the equation E_O_ = k_O_ / (k_O_ + k_C_), and the time constant of opening and closing (τ) is given by the equation τ  = (k_O_ + k_C_)^-1^. Experimental E_O_ and τ  values were plotted against the membrane potential, and the plots were approximated by adjusting the three – three parameters for k_O_ and k_C_.

Although for the MSA model we could start from the parameters of the model described by Kuo and Bean [6], which provided adequate results, we had to introduce slow-inactivated states, which altered the behavior of the whole model, including activation and fast inactivation. Therefore, we first adjusted the parameters manually, as described above for the tetracube model.

**Optimization:** We used the Nelder-Mead method for optimization [14]. The optimization program was written in C++. In the case of the tetracube and MSA models, we had 18 and 20 free parameters, respectively. Accuracy of fit was evaluated based on the five curves shown in Figure S1, and for each curve deviations at 10 to 20 points were calculated. In the case of the steady-state inactivation and activation curves (Figure S1A), for the sake of accuracy, we used data collected from outside-out macropatches. Measurements from four individual cells were fitted with Boltzmann functions, and the parameters of the Boltzmann functions were averaged. For fitting, we used the curve that was reconstructed using the mean of the parameters. For the “SInact_t” and “Rec_t” curves (Figure S1B), we used averaged whole-cell measurements (as shown in Figures 5B and C in the paper). For evoked current (Figure S1C), we used a single recording from one of the outside-out macropatches.

Results of the optimization were evaluated and then manually adjusted again before a second cycle of optimization. The final parameters and limits for optimization are shown in Table S3 for the tetracube model, and in Table S4 for the MSA model. From the illustration of measured and simulated curves (Figure S1), the limitations of both models can be seen. First, as we have discussed, the tetracube model was unable to reproduce current activation kinetics. Another limitation of the tetracube model is that the activation curve decreases at positive membrane potentials. (These shortcomings are of no importance regarding our current questions, because we are interested in availability at different times, not the exact shape of the currents.) The main limitation of the MSA model was that it could not reproduce the slope of the inactivation curve. This probably is the consequence of the fact that, unlike in real channels, voltage sensors are equivalent and independent in this model.

**Drug simulations with MSA model compared to the tetracube model**

We repeated the simulations using the MSA model with the prototypical drugs discussed in the paper and shown in Figure 2 (Figure S2). The main differences between the two models were: i) At -60 mV the current was missing altogether because of the steep steady-state inactivation curve (see Figure S1A) and, therefore, we show the concentration response curve at the -70 mV holding potential (Figure S2A); ii) “SI” drugs were more potent even at -150 mV; iii) The leftward shift of inactivation curves was much smaller; and iv) Steady-state inactivation curves were steeper (Figure S2B, for MSA model, Figure 2B for tetracube model, see also Figure 5A for experiments). On the other hand, the effects of the “drugs” in the “SInact_t” and “Rec_t” protocols were qualitatively identical with the data obtained using the tetracube model (Figure S2C-D.).

Despite the differences between the two models, the three major observations are the same in both models: 1) A shift of the “SInact_V” curve does not necessarily reflect a slow-inactivated state preference; 2) Fast-inactivated state-stabilizing drugs caused a larger shift of the “SInact_t” curve than slow-inactivated state-preferring drugs; and 3) Slow-inactivated state-preferring drugs are not necessarily more effective than fast-inactivated state-preferring ones in delaying recovery in the “Rec_t” protocol.

**References**

1. Hodgkin AL, Huxley AF (1952) A quantitative description of membrane current and its application to conduction and excitation in nerve. J Physiol 117: 500-544.

2. Vedantham V, Cannon SC (1999) The position of the fast-inactivation gate during lidocaine block of voltage-gated Na+ channels. J Gen Physiol 113: 7-16.

3. Wang SY, Mitchell J, Moczydlowski E, Wang GK (2004) Block of inactivation-deficient Na+ channels by local anesthetics in stably transfected mammalian cells: evidence for drug binding along the activation pathway. J Gen Physiol 124: 691-701.

4. Sheets MF, Hanck DA (2007) Outward stabilization of the S4 segments in domains III and IV enhances lidocaine block of sodium channels. J Physiol 582: 317-334.

5. Balser JR, Nuss HB, Orias DW, Johns DC, Marban E, et al. (1996) Local anesthetics as effectors of allosteric gating. Lidocaine effects on inactivation-deficient rat skeletal muscle Na channels. J Clin Invest 98: 2874-2886.

6. Kuo CC, Bean BP (1994) Na+ channels must deactivate to recover from inactivation. Neuron 12: 819-829.

7. Patlak J (1991) Molecular kinetics of voltage-dependent Na+ channels. Physiol Rev 71: 1047-1080.

8. Ulbricht W (2005) Sodium channel inactivation: molecular determinants and modulation. Physiol Rev 85: 1271-1301.

9. Vandenberg CA, Bezanilla F (1991) A sodium channel gating model based on single channel, macroscopic ionic, and gating currents in the squid giant axon. Biophys J 60: 1511-1533.

10. Vandenberg CA, Horn R (1984) Inactivation viewed through single sodium channels. J Gen Physiol 84: 535-564.

11. Aldrich RW, Stevens CF (1987) Voltage-dependent gating of single sodium channels from mammalian neuroblastoma cells. J Neurosci 7: 418-431.

12. Mike A, Karoly R, Vizi ES, Kiss JP (2003) Inhibitory effect of the DA uptake blocker GBR 12909 on sodium channels of hippocampal neurons. Neuroreport 14: 1945-1949.

13. O'Leary ME (1998) Characterization of the isoform-specific differences in the gating of neuronal and muscle sodium channels. Can J Physiol Pharmacol 76: 1041-1050.

14. Nelder JA, Mead R (1965) A Simplex Method for Function Minimization. The Computer Journal 7: 308-313.
